# Life Insurance Explained

**Published:** 1852-01

**Authors:** 


LIFE INSURANCE EXPLAINED.

We have received the first number of a periodical, devoted to the explanation of Life Insurance. The importance of the subject, the deep interest which all should feel in it, and the necessity that exists for a thorough understanding of all questions connected with Life Insurance should command a great circulation for this new periodical. There are few, even of those who feel interest enough to take out a policy on their lives, who acquaint themselves with much more on the subject than the amount for which they insure, and the premiums that are to be paid on the policy. The questions connected with life insurance are varied in their character, and require to be well studied and well understood. For instance; there are two kinds of Life Insurance agencies. In one the premiums are required to be paid in cash; in the other, a portion is paid in cash, and a credit secured by notes, is given. Any man of sound reflection must see that there is a material difference between these two kinds of insurance. One must of necessity be much more secure and prosperous than the other. The same principle holds good in these Insurances that prevails in other things. A business based on a cash capital, other things being equal, must be more secure and prosperous than that which works upon credit. There are a great variety of causes that speak loudly of the superior advantages of the Insurance based on cash payments, but we have not time nor space to dilate upon them.

The periodical to which we have referred, is published in Philadelphia, bi-monthly, and will consist of a series of six lectures, at fifty cents, if paid in advance. The work is edited by M. L. Knapp, M. D., and, judging from one number of the work, the editor has ample capacity for his enterprise. We wish him a great deal of success in his laudable undertaking. B.

